# A simple protocol for the subcellular fractionation of skeletal muscle cells and tissue

**DOI:** 10.1186/1756-0500-5-513

**Published:** 2012-09-20

**Authors:** Ivan Dimauro, Timothy Pearson, Daniela Caporossi, Malcolm J Jackson

**Affiliations:** 1 Department of Musculoskeletal Biology, Institute of Ageing & Chronic Disease, University of Liverpool, Daulby Street, Liverpool L69 3GA, United Kingdom; 2 Department of Health Sciences, University of Rome “Foro Italico”, Piazza Lauro De Bosis 15, 00194, Rome, Italy

**Keywords:** Skeletal muscle, Subcellular fractionation, Western blotting

## Abstract

**Background:**

We describe a method for subcellular fractionation of mouse skeletal muscle, myoblast and myotubes to obtain relatively pure fractions of nuclear, cytosolic and mitochondrial compartments. Fractionation allows the analysis of a protein of interest (or other cellular component) based on its subcellular compartmental distribution and can also generate molecular information about the state of a cell and/or tissue and how the distribution of a protein may differ between different cellular compartments, tissues or cell types, in response to treatments or ageing.

**Findings:**

The described method was specifically developed for skeletal muscle and proliferating/differentiated muscle cells. The purity of the different fractions, representing the cytoplasmic, mitochondrial and nuclear subcellular compartments was validated by western blot analysis of “house-keeper” marker proteins specific for each cellular compartment.

**Conclusion:**

This low cost method allowed the mitochondrial, cytoplasmic and nuclear subcellular compartments from the same starting muscle samples to be rapidly and simultaneously isolated with good purity and without the use of an ultracentrifuge. This method permits samples to be frozen at −80°C for future analysis and/or additional processing at a later date.

## Findings

### Background

Isolation of nuclear, cytosolic and mitochondrial fractions of reasonable purity from mammalian tissues and cells has generated great interest as it has the advantage of allowing different cellular proteins and organelles to be studied and characterised. Subcellular fractionation is universally used for various cell types and tissues for sample preparation and prior to subsequent ~ omics analysis [1-5]. Generic fractionation protocols exist that can purify specific subcellular compartments and organelles, but in general they are not tailored for use with skeletal muscle and may require large amounts of starting material, time, or special reagents whilst potentially yielding fewer fractions from the same starting sample etc. [3,5-9]. The protocol described has been optimized for use with primary skeletal muscle tissue (e.g. mouse *anterior tibialis* (AT) muscle) and both proliferating and differentiated C2C12 cells to isolate subcellular fractions of nuclei, cytosol, and mitochondria from a single starting sample, thereby reducing the quantity of starting material, cost and total time needed for sample preparation.

The protocol works well for skeletal muscle tissue and cells and could be used as a starting point for the fractionation of other non-muscle samples although changes to buffer volumes; homogenization duration/intensity etc. may be required. The purity of the fractions obtained was assessed by immunoblotting for specific protein markers: histone H3 (nuclei), glyceraldehyde 3-phosphate dehydrogenase (GAPDH, cytosol), and cytochrome oxidase IV (CoxIV, mitochondria).

### Cell culture and animals

The C2C12 mouse skeletal myoblast cell line was obtained from the American Type Culture Collection (CRL-1772). C2C12 myoblasts were maintained in DMEM (Sigma Aldrich, Poole, UK) supplemented with 1% L-glutamine (Lonza, Cologne, Germany), 10% FBS (Biosera, Sussex, UK) and 1% penicillin and streptomycin (Sigma) under an atmosphere of 5% CO_2_ in humidified air at 37°C. To induce myogenic differentiation, the growth medium was changed to differentiation medium (DMEM supplemented with 2% horse serum (Sigma) and 1% antibiotics) after myoblasts had reached ≈ 90% confluence in a T75 cm^2^ flask. Myoblast cells were either harvested at 90% confluence or allowed to mature to myotubes for 7 days and then harvested (see below).

Adult mice (C57BL/6) were euthanized by overdose with anesthetic (ketamine hydrochloride and medatomidine hydrochloride) administered by intraperitoneal injection. *Anterior tibialis* (AT) muscles, approximately 50 mg wet weight, were rapidly removed and used fresh to prepare fractions. Experiments were performed in accordance with UK Home Office Guidelines under the UK Animals (Scientific Procedures) Act 1986 and received ethical approval from the University of Liverpool Animal Welfare Committee.

### Subcellular fractionation

**Figure 1:**
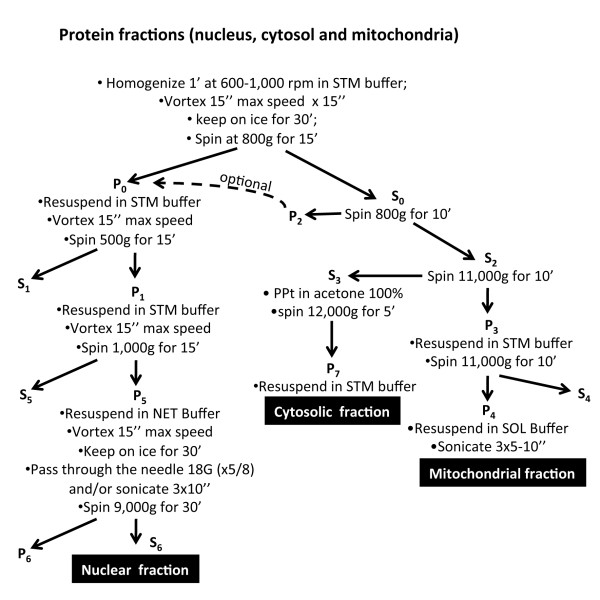
**Schematic representation of the fractionation protocol. **The developed protocol provides three subcellular fractions of cytoplasm, nuclei and mitochondria from a muscle sample. (− − −) Dotted arrow shows an optional step.

Fresh AT tissue and scraped cells were washed with cold PBS, cells were pelleted by centrifugation at 200 *g* for 7 minutes whereas tissues were placed in a pre-chilled glass Petri dish and minced on ice using sharp scissors. All samples were resuspended in 300-500 μl of STM buffer comprising 250 mM sucrose, 50 mM Tris–HCl pH 7.4, 5 mM MgCl_2_, protease and phosphatase inhibitor cocktails (all chemicals were from Sigma-Aldrich, Poole, UK unless stated otherwise) and homogenized for 1 minute on ice using a tight-fitting Teflon pestle attached to a Potter S homogeniser (Sartorius Stedium, Goettingen, Germany) set to 600–1,000 rpm. The homogenate was then inspected, if intact tissue was still evident the homogenisation was repeated. The homogenate was decanted into a centrifuge tube and maintained on ice for 30 minutes, vortexed at maximum speed for 15 seconds and then centrifuged at 800 *g* for 15 minutes. The pellet was labelled as P_0_ and kept on ice, the supernatant was labelled as S_0_ and used for subsequent isolation of mitochondrial and cytosolic (Figure 1) fractions.

The pellet P_0_ (containing nuclei and debris) was resuspended in 300-500 μl STM buffer, vortexed at maximum speed for 15 seconds and then centrifuged at 500 *g* for 15 minutes. Following the above step, the nuclear pellet was labelled as P_1_ and kept on ice, the supernatant S_1_ (cell debris) was discarded. The purity of the nuclei within fraction P1 can be quickly determined by microscopic inspection by diluting an aliquot of the fraction in a trypan blue solution on a haemocytometer. If the P_1_ fraction contained excess cell debris the above step was repeated once.

To increase the P_1_ fraction purity further it was washed in STM buffer (300-500 μl), vortexed at maximum speed for 15 seconds and then centrifuged at 1,000 *g* for 15 minutes. The washed pellet was labelled as P_5_ (S_5_ was discarded) and resuspended in 200-500 μl NET buffer (comprising: 20 mM HEPES pH 7.9, 1.5 mM MgCl_2_, 0.5 M NaCl, 0.2 mM EDTA, 20% glycerol, 1% Triton-X-100, protease and phosphatase inhibitors) using a pipette to triturate until homogeneous. Pellet P_5_ was vortexed at maximum speed for 15 seconds and incubated on ice for 30 minutes, this fraction contained the nuclei. The nuclei were lysed with 10–20 passages through an 18-gauge needle and/or sonicated (using a Soniprep 150, MSE, London, UK) at high setting for 10–15 seconds with 30 second pauses whilst being kept on ice throughout. The lysate was centrifuged at 9,000 *g* for 30 minutes (at 4°C), the resultant supernatant (S_6_) was the final “nuclear fraction” (Figure 1).

Cytosolic and mitochondrial fractions were extracted from S_0_ by centrifugation at 800 *g* for 10 minutes. The supernatant S_2_ was saved and the pellet (P_2_) was discarded, though to improve the nuclear yield the pellet P_2_ can be combined with fraction P_0_ (optional step). S_2_ was then centrifuged at 11,000 *g* for 10 minutes and the supernatant S_3_ (containing cytosol and microsomal fraction) was precipitated in cold 100% acetone at −20°C for at least 1 hour followed by centrifugation at 12,000 *g* for 5 minutes, the pellet (P_7_) was then resuspended in 100-300 μl STM buffer and labelled as “cytosolic fraction” (Figure 1) that likely included some microsomal content. The pellet P_3_ was again resuspend in 100-200 μl STM buffer and centrifuged at 11,000 *g* for 10 minutes. Once centrifuged, supernatant S_4_ was discarded, the mitochondrial pellet (P_4_) was resuspended in 50-100 μl SOL buffer (comprising: 50 mM Tris HCl pH 6.8, 1 mM EDTA, 0.5% Triton-X-100, protease and phosphatase inhibitors) by sonication on ice at high setting for 5–10 seconds with 30 second pauses and labelled as “mitochondrial fraction”. All buffers and centrifugation steps were modified from Cox and Emili [9] and Psarra et al. [10].

Additionally, a commercial cell fractionation kit designed to yield near pure nuclei and cytoplasmic fractions from the same starting sample of cells and soft tissues was used (Thermo NE-PER nuclear and cytoplasmic extraction kit, Pierce-Thermo, Northumberland, UK, to obtain a mitochondrial fraction required the use of an additional kit) as directed by the manufacturer. This enabled a comparison of the efficacy of the commercial kit with the method described here when both methods were used to fractionate an identical starting sample (myoblast cells from a T75 cm^2^ flask, approximately 2×10^6^ cells).

The protein content of each compartment was determined using BCA protein assay (Sigma).

### Fractionation validation using western blotting

**Figure 2:**
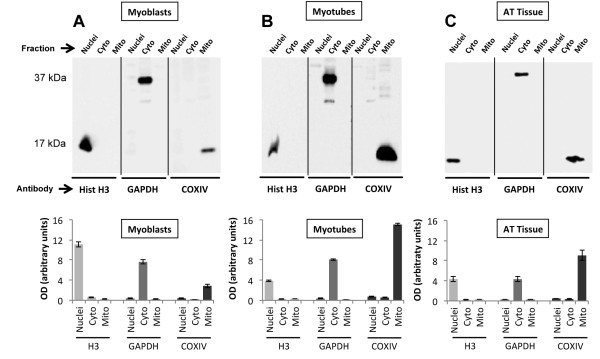
**Example western blots from three starting samples. **In the top panel; (**A**) myoblast sample (20 μg protein was added to each lane) fractionated into nuclei, cytoplasmic and mitochondrial samples. Each fraction was run side-by-side on the same blot and then probed separately against each of the three primary antibodies, Histone-H3, GAPDH and CoxIV, to validate fraction purity. This was repeated for; (**B**) myotubes (20 μg protein per lane) and (**C**) AT tissue (15 μg protein per lane). In the bottom panel; marker protein fraction expression was measured as OD per resultant band area and was expressed in arbitrary units. The histogram data are representative of the mean (± SEM) of five separate experiments.

Validation of the purity of the subcellular fractions derived from the same starting sample was determined by examining “house-keeper” (HK) protein markers by standard SDS-PAGE analysis. Protein samples (15–20 μg/fraction) were separated by a 12% SDS/PAGE and transferred onto a nitrocellulose membrane (Sigma) and probed using monoclonal antibodies for Histone H3 (Cell Signalling, Hertfordshire, UK, 1: 2,000), GAPDH (Abcam, Cambridge, UK, 1: 5,000), and Cox IV (Abcam, Cambridge, UK, 1: 2,000) for nuclei, cytosolic and mitochondrial HK fractions respectively. Proteins were then visualized after applying specific secondary HRP-conjugated antibodies and exposure to a supersignal west dura substrate (Pierce-Thermo, Northumberland, UK) by use of a ChemiDoc^TM^ XRS (Bio-Rad, Hertfordshire, UK) see Figure 2 (representative example of at least three replicates per sample). Secondary antibody controls were also investigated and no non-specific binding was apparent (data not shown). To assess the purity of each fraction, bands relative to sub-compartment protein marker were quantified by ImageJ software [11].

## Results and discussion

After fractionation and extraction of proteins from the C2C12 cell cultures and AT muscles using the protocol described in Figure 1, we report the mean fraction yield values obtained from replicate experiments (n = 5) (Table 1). Figure 2A-C show the nuclear, cytoplasmic and mitochondrial fractions obtained from a single starting sample from each of the different tissue/cell types. The fractions were examined by western blotting analysis using antibodies directed against specific HK markers and revealed single dense bands for histone H3 at approximately 17 kDa in the nuclear fraction, a band representing GAPDH at approximately 37 kDa in the cytosolic fraction, and a single band at approximately 17 kDa for CoxIV in the mitochondrial fraction (Figure 2A-C) in each sample.

**Table 1:** Fraction yields for myoblast, myotube and AT tissue

**Sample**	**Mean protein yield (μg)**			**Total sample protein (mg)**	**No. of replicates**
	**Nuclei**	**Cyto**	**Mito**		
**Myoblast**	1179 ± 100 (150μL)	496 ± 25 (100μL)	97 ± 13 (50μL)	1.8 ± 0.1	5
**Myotube**	1624 ± 107 (150μL)	547 ± 4 (100μL)	197 ± 15 (50μL)	2.4 ± 0.2	5
**AT tissue**	3294 ± 254 (300μL)	1486 ± 225(300μL)	444 ± 60 (100μL)	5.2 ± 0.5	5

Typical protein yield (μg) obtained after sample fractionation using the indicated volume of lysis buffer for myoblasts, myotubes (both cultured in 75 cm^2^ flask, ≈2×10^6^ cells) and AT muscle (all n = 5, mean ± SEM). The protein yields (determined by BCA assay as *total* volume of a fraction × protein concentration in the fraction) are representative and dependent upon the volume of buffer utilized to resuspend each fraction.

These analyses showed little contamination between compartment fractions indicating that the purities of the nuclear, cytosolic, and mitochondrial fractions were relatively high (Table 2). The HK band intensity was high arguing that enrichment of proteins was achieved during processing (Table 1). The nuclear and mitochondrial fractions showed the highest band density indicating good yields and the lack of equivalent molecular weight bands in other fractions argued relatively little cross-talk and indicated reasonable purity, with the myotubes being slightly more variable depending on the fraction (feint additional non-specific bands were evident in the cytosolic fraction from myotubes). Furthermore, analysis of the distribution of the sub-compartment fraction marker proteins (Table 2) showed the minimum percentage of purity was ≈ 85.6% (Cox IV mitochondrial marker in myoblasts) and therefore argued less than 15% was from other sources.

**Table 2:** Purity analysis of the yield of sub-compartmental marker proteins

**Fraction/Marker**	**Myoblasts**	**Myotubes**	**AT Tissue**
**Nuclei/Histone H3 (%)**	92.7 ± 1.3	86.4 ± 1.4	88.0 ± 0.9
**Cyto/GAPDH (%)**	92.4 ± 0.5	93.9 ± 1.4	87.8 ± 0.3
**Mito/CoxIV (%)**	85.6 ± 0.9	92.1 ± 0.5	91.2 ± 0.1

The percentage shown in the table is obtained by the ratio between OD values of Histone H3, GAPDH and CoxIV (all n = 5, mean ± SEM) in an individual target compartment divided by the sum of the OD values present in all compartments in each sample.

**Figure 3:**
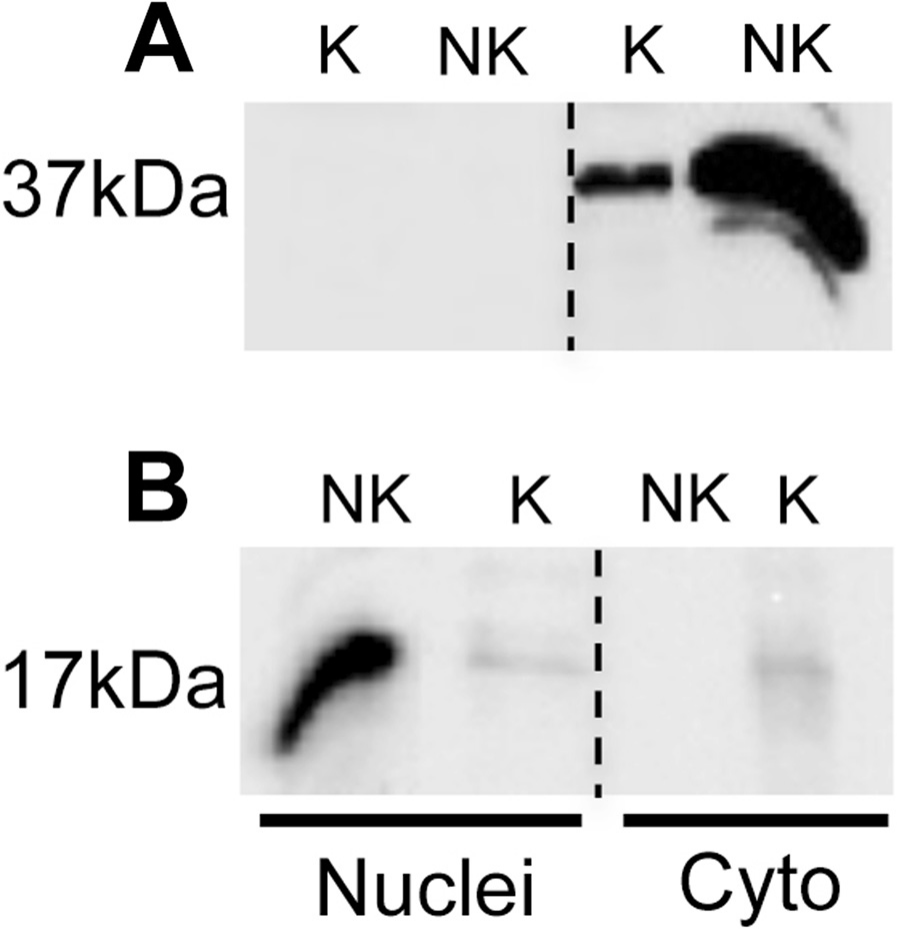
**Comparison of fractions generated from a muscle source by a kit versus the described method.** Example western blot to compare sample purity generated after using a commercial cell fractionation kit (K = kit) versus the method reported here (NK = not kit). The kit was used to generate nuclear and cytoplasmic fractions only, these were compared for purity and yield against the method described by probing both fractions for (**A**) GAPDH and (**B**) Histone-H3. The C2C12 myotube fractions obtained from both methods were analysed on the same western blot (20 μg protein was loaded from each fraction).

Comparison of the method described with a commercially available fractionation kit showed both approaches generated clean and abundant cytoplasmic fractions (Figure 3A), although the yield using the kit was smaller. The nuclear fraction generated by the kit showed both reduced yield and compromised purity (Figure 3B). The kit was not designed to generate a mitochondrial fraction and so no equivalent comparison was undertaken.

## Conclusion

In comparison with the described protocol, the yield obtained with the commercial kit as indicated by band density, was poor and only the cytoplasmic fraction appeared relatively pure. The kit is designed for more generic use with multiple cells and soft tissues and may explain the inability to generate a pure and abundant nuclear fraction from muscle. The main advantage of the kit was a slightly reduced processing time although an additional kit to purify a mitochondrial (and cytoplasmic) fraction would be required and this would increase the cost and time requirement.

In conclusion the method for subcellular fractionation described here is inexpensive, does not require an ultracentrifuge and was found to generate three relatively abundant subcellular fractions of reasonable purity. This method of subcellular fractionation could be combined with proteomics research wherein protein patterns of subcellular fractions could be mapped and characterized by 2D gel analysis and mass spectrometry.

## Competing interests

The authors declare that they have no competing interests.

## Authors’ contributions

ID and TP designed and carried out the cell culture and tissue experiments; MJJ and TP developed and supervised the experiments; MJJ, ID, TP and DC interpreted the data and wrote the manuscript. All authors read and approved the final manuscript.

## Authors’ information

Ivan Dimauro and Timothy Pearson joint first author.

## References

[B1] De Duve C Pressman BC Gianetto R Wattiaux R Appelmans F Tissue fractionation studies. 6. Intracellular distribution patterns of enzymes in rat-liver tissue Biochem J 1955 60 604 617 13249955 10.1042/bj0600604 PMC1216159

[B2] Bronfman M Loyola G Koenig CS Isolation of intact organelles by differential centrifugation of digitonin-treated hepatocytes using a table Eppendorf centrifuge Anal Biochem 1998 255 252 256 10.1006/abio.1997.2453 9451511

[B3] Srinivas KS Chandrasekar G Srivastava R Puvanakrishnan R A novel protocol for the subcellular fractionation of C3A hepatoma cells using sucrose density gradient centrifugation J Biochem Biophys Methods 2004 60 23 27 10.1016/j.jbbm.2004.04.011 15236907

[B4] Kislinger T Cox B Kannan A Chung C Hu P Ignatchenko A Scott MS Gramolini AO Morris Q Hallett MT Rossant J Hughes TR Frey B Emili A Global survey of organ and organelle protein expression in mouse: combined proteomic and transcriptomic profiling Cell 2006 125 173 186 10.1016/j.cell.2006.01.044 16615898

[B5] Holden P Horton WA Crude subcellular fractionation of cultured mammalian cell lines BMC Res Notes 2009 2 243 10.1186/1756-0500-2-243 20003239 PMC2802353

[B6] Mootha VK Bunkenborg J Olsen JV Hjerrild M Wisniewski JR Stahl E Bolouri MS Ray HN Sihag S Kamal M Patterson N Lander ES Mann M Integrated analysis of protein composition, tissue diversity, and gene regulation in mouse mitochondria Cell 2003 115 629 640 10.1016/S0092-8674(03)00926-7 14651853

[B7] Krapfenbauer K Fountoulakis M Lubec G A rat brain protein expression map including cytosolic and enriched mitochondrial and microsomal fractions Electrophoresis 2003 24 1847 1870 10.1002/elps.200305401 12783461

[B8] Dreger M Bengtsson L Schöneberg T Otto H Hucho F Nuclear envelope proteomics: novel integral membrane proteins of the inner nuclear membrane Proc Natl Acad Sci U S A 2001 98 11943 11948 10.1073/pnas.211201898 11593002 PMC59747

[B9] Cox B Emili A Tissue subcellular fractionation and protein extraction for use in mass-spectrometry-based proteomics Nature Protocols 2006 4 1872 1878 10.1038/nprot.2006.273 17487171

[B10] Psarra AM Solakidi S Trougakos I Margaritis L Spyrou G Sekeris C Glucocorticoid receptor isoforms in human hepatocarcinoma HepG2 and SaOS-2 osteosarcoma cells: Presence of gluocorticoid receptor alpha in mitochondria and of glucocorticoid receptor beta in nucleoli The Int. Jnl. Biochem. & Cell Biol 2005 37 2544 2558 10.1016/j.biocel.2005.06.015 16076561

[B11] Rasband WS Image J US National Institutes of Health 1997–2008 Bethesda, Maryland, USA http://rsb.info.nih.gov/ij

